# Multiparametric MRI and Whole Slide Image-Based Pretreatment Prediction of Pathological Response to Neoadjuvant Chemoradiotherapy in Rectal Cancer: A Multicenter Radiopathomic Study

**DOI:** 10.1245/s10434-020-08659-4

**Published:** 2020-07-29

**Authors:** Lizhi Shao, Zhenyu Liu, Lili Feng, Xiaoying Lou, Zhenhui Li, Xiao-Yan Zhang, Xiangbo Wan, Xuezhi Zhou, Kai Sun, Da-Fu Zhang, Lin Wu, Guanyu Yang, Ying-Shi Sun, Ruihua Xu, Xinjuan Fan, Jie Tian

**Affiliations:** 1 School of Computer Science and Engineering, Southeast University, Nanjing, China; 2 CAS Key Laboratory of Molecular Imaging, Institute of Automation, Beijing, China; 3 School of Artificial Intelligence, University of Chinese Academy of Sciences, Beijing, China; 4 Department of Radiation Oncology, The Sixth Affiliated Hospital of Sun Yat-sen University, Guangzhou, China; 5 Department of Pathology, The Sixth Affiliated Hospital of Sun Yat-sen University, Guangzhou, China; 6 Department of Radiology, Yunnan Cancer Center, Yunnan Cancer Hospital, The Third Affiliated Hospital of Kunming Medical University, Kunming, China; 7 Key Laboratory of Carcinogenesis and Translational Research (Ministry of Education/Beijing), Department of Radiology, Peking University Cancer Hospital & Institute, Beijing, China; 8 Engineering Research Center of Molecular and Neuro Imaging of Ministry of Education, School of Life Science and Technology, Xidian University, Xi’an, China; 9 Department of Pathology, Yunnan Cancer Center, Yunnan Cancer Hospital, The Third Affiliated Hospital of Kunming Medical University, Kunming, China; 10 LIST, Key Laboratory of Computer Network and Information Integration, Southeast University, Ministry of Education, Nanjing, China; 11 State Key Laboratory of Oncology in South China, Collaborative Innovation Center for Cancer Medicine, Sun Yat-sen University Cancer Center, Guangzhou, China; 12 Beijing Advanced Innovation Center for Big Data-Based Precision Medicine, School of Medicine, Beihang University, Beijing, China

## Abstract

**Background:**

The aim of this work is to combine radiological and pathological information of tumor to develop a signature for pretreatment prediction of discrepancies of pathological response at several centers and restage patients with locally advanced rectal cancer (LARC) for individualized treatment planning.

**Patients and Methods:**

A total of 981 consecutive patients with evaluation of response according to tumor regression grade (TRG) who received nCRT were retrospectively recruited from four hospitals (primary cohort and external validation cohort 1–3); both pretreatment multiparametric MRI (mp-MRI) and whole slide image (WSI) of biopsy specimens were available for each patient. Quantitative image features were extracted from mp-MRI and WSI and used to construct a radiopathomics signature (RPS) powered by an artificial-intelligence model. Models based on mp-MRI or WSI alone were also constructed for comparison.

**Results:**

The RPS showed overall accuracy of 79.66–87.66% in validation cohorts. The areas under the curve of RPS at specific response grades were 0.98 (TRG0), 0.93 (≤ TRG1), and 0.84 (≤ TRG2). RPS at each grade of pathological response revealed significant improvement compared with both signatures constructed without combining multiscale tumor information (*P* < 0.01). Moreover, RPS showed relevance to distinct probabilities of overall survival and disease-free survival in patients with LARC who underwent nCRT (*P* < 0.05).

**Conclusions:**

The results of this study suggest that radiopathomics, combining both radiological information of the whole tumor and pathological information of local lesions from biopsy, could potentially predict discrepancies of pathological response prior to nCRT for better treatment planning.

**Electronic supplementary material:**

The online version of this article (10.1245/s10434-020-08659-4) contains supplementary material, which is available to authorized users.

Standard treatment for locally advanced rectal cancer (LARC) includes neoadjuvant chemoradiotherapy (nCRT) followed by total mesorectal excision (TME) and adjuvant chemotherapy,^1^,^2^ which is able to decrease the local relapse, downstage and downsize the tumor, and increase the rates of subsequent successful R0 resection and sphincter-preserving surgery.^3^ Patients’ pathological response to nCRT is usually evaluated by tumor regression grade (TRG) as TRG0: no remaining viable cancer cells; TRG1: only small clusters or single cancer cells remaining; TRG2: residual cancer remaining, but with predominant fibrosis; and TRG3: minimal or no tumor kill with extensive residual cancer, combined with lymph node status, which is variable and available only after completion of all preoperative treatment and surgery and thus cannot provide guidance for adjusting the therapeutic approach. Importantly, TRG has been related to distinct probabilities of cancer recurrence and survival, especially between TRG0 and TRG3.^4^–^6^ After nCRT, 15–27% of patients with LARC who receive nCRT will achieve pathologic complete response (pCR), usually achieving perfect long-term outcomes, preferring to avoid surgery, and undergoing an organ-preserving strategy such as “watch and wait” management.^7^–^9^ Additionally, for the more than 50% of patients who are unable to reach good response (GR),^10^ treatment optimization according to different pathological responses is essential to balance the benefits of nCRT versus its toxicity.^11^ Therefore, it is important to establish a reliable signature for predicting response discrepancies prior to nCRT.

Multiparametric magnetic resonance imaging (mp-MRI) is a requisite examination for LARC. Several investigators have revealed that radiomics using quantitative and high-dimensional image features from mp-MRI and the machine learning method provided a new strategy for cancer diagnosis, efficacy evaluation, and prognosis.^12^–^14^ Radiomics using pre- and posttreatment mp-MRI showed the possibility of evaluating pCR after nCRT to distinguish patients who could avoid TME.^15^ Furthermore, several previous studies have predicted that response to nCRT can guide therapy regimen planning, not only helping patients to avoid surgery but also as a pretreatment prediction of pCR and GR with two binary category models.^16^,^17^ Some works have combined this with more imaging modalities to construct a better prediction model by enriching the tumor description.^18^ Nevertheless, personalized treatment still requires a more hierarchical prediction model based on different pathological responses, to reduce decision costs. More importantly, all previous studies only considered the tumor on a macroscale, using medical imaging to depict the whole tumor. This may suffer from the potential risk of overlooking tumor heterogeneity. In some cases, compared with macroscopic information from mp-MRI of whole tumor, pathological information from microscopic observation is necessary to enrich the description of lesions, but insufficient attention has been paid to the efficacy of such correlations.

Pathological evaluation of the biopsy specimen from colonoscopy is the gold standard for rectal cancer diagnosis. Based on existing reports, this shows great application prospects for predictions of curative effect and prognosis in rectal cancer.^19^,^20^ Besides, compared with conventional pathological diagnosis by visual evaluation of morphology and grade differentiation, the subvisual morphometric phenotypes of the digitizing whole slide image (WSI) mined by machine learning provides a digital tool for pathological evaluation of the biopsy specimen, possibly having promising applications in treatment optimization.^21^ Meanwhile, it may be possible to integrate features from WSI with gene information to construct a better prediction model for prognosis.^22^ It is hoped that combining radiological information of whole tumor at macroscale and pathological information of local lesions at microscale to enrich the tumor descriptors will provide prospects for the construction of a more powerful model for predicting tumor response to nCRT.

To design a more reliable signature to predict discrepancies of pathological response prior to nCRT, for better treatment planning and validation in multicenter datasets, a new strategy named radopathomics with both mp-MRI and WSI, which takes advantage of combined tumor information at different scales, is proposed herein.

## Patients and Methods

### Patients

A total of 981 patients treated with nCRT between May 2007 and November 2017 at four hospitals specializing in gastrointestinal disease in China [the Sixth Affiliated Hospital of Sun Yat-sen University (SYSU6, *N* = 303), Sun Yat-sen University Cancer Center (SYSUCC, *N* = 480), Yunnan Cancer Hospital (YNCH, *N* = 150), and Peking University Cancer Hospital (PUCH, *N* = 48)] were retrospectively recruited (Fig. S1). The inclusion criteria were as follows: (1) All patients underwent pretreatment MRI and biopsy under electronic colonoscopy with hematoxylin–eosin (H&E) staining biopsy section digitalized to a WSI and were defined as LARC (cT3–4/N0–2/M0); (2) All patients underwent nCRT (Supplementary Information SI); (3) All patients underwent standard TME surgery after nCRT; (iv) Pathological response was confirmed by experienced pathologists using a four-category AJCC/CAP TRG system after TME surgery (Supplementary Information SII). The exclusion criteria were as follows: (1) Patient accepted nonstandard or incomplete nCRT or had neoadjuvant chemo- or radiotherapy alone; (2) Treatment response data of patient were unavailable; (3) Lack of biopsy pathological slides or quality of WSI did not meet the requirements for diagnosis (e.g., tissue folds, torn tissue); (4) Lack of pretreatment T2-weighted MRI (T2WI), diffusion-weighted imaging (DWI), or insufficient quality of MRI images to obtain measurements (e.g., due to motion artifacts). This multicenter study was conducted in accordance with the Declaration of Helsinki and was approved by the ethics committee of the Sixth Affiliated Hospital of Sun Yat-sen University (approval no. 2019ZSLYEC-169), with the requirement for informed consent waived. Patients enrolled from SYSU6 with the same magnetic resonance (MR) acquisition parameters were used as the primary cohort (PC) to reduce any form of overfitting or bias in the analysis, while the other three datasets (SYSUCC, YNCH, and PUCH) were used as independent validation cohorts (VC1–VC3).

### Acquisition and Annotation of Images

Rectal MR examination for each patient was performed before biopsy and within 1–2 weeks before nCRT treatment. Axial fat-suppressed T2WI and DWI with two *b* values (0 and 1000 s/mm^2^ or 800 s/mm^2^) were acquired for each patient using 1.5-T or 3.0-T scanners at the four hospitals (Table S1). The regions of interest (ROIs) of tumors in the MRI images were manually annotated using itk-SNAP software (www.itksnap.org) on each slice (Supplementary Information SIII, Fig. S2).

H&E-stained slides from biopsy formalin-fixed paraffin-embedded (FFPE) tissue were used for pathological diagnosis. WSI for analysis was collected by panoramic digital image scanning technology using a Leica AT2 or CS2 at each institution. The ROIs of tumor in biopsy WSI were manually delineated using the ImageScope software (www.leicabiosystems.com) at 10 × magnification (1 µm/pixel) by four expert pathologists and rechecked at 20 × magnification (0.5 µm/pixel) by another two expert pathologists to ensure boundary validity (Fig. S3).

### Features Extraction from mp-MRI

MRI of each patient was normalized with *Z*-scores to obtain a standard normal distribution of image intensities. Next, a total of 702 quantitative image features, named radiomic features, were extracted from the normalized pretreatment T2WI and apparent diffusion coefficient (ADC) data with manually segmented ROIs by Pyradiomics (version 2.1.1, https://github.com/Radiomics/pyradiomics) using Python.^23^ Intraclass correlation coefficients (ICCs) were utilized to evaluate the intra- and interobserver agreement in terms of feature extraction and reducing the negative impact of manual segmentation on the extracted features. Details of extracted radiomic features are illustrated in Supplementary Information SIV.

### Feature Extraction from WSI

Features were extracted by automated histopathological imaging analysis systems to represent the levels of tumor cell differentiation, i.e., the pathological level information, named pathomic features. A total of 770 features, including pixel intensity, morphology, and nuclear texture for each ROI, were extracted using CellProfiler platform (version 2.2.1, https://cellprofiler.org/),^23^ an open-source tool widely used in the field of biological image analysis. Details of the extracted pathomic features are illustrated in Supplementary Information SV.

### Signature Construction and Validation

A radiopathomics signature (RPS) was constructed using both radiomic and pathomics features within the PC. The eXtreme Gradient Boosting algorithm (XGBoost, https://github.com/dmlc/xgboost) was used to select useful features from a pool of radiomic and pathomic features and build a prediction model for different response.^24^ The metric for feature selection was the average gain of the feature (Gain), evaluating the importance of the feature during feature screening. Satisfactory features with Gain > 0 were recorded as radiopathomic features. Then, the Spearman test was used to evaluate the significance between each of the recorded features and true response levels and eliminate the correlation values without statistical significance (*P* < 0.05) to further optimize the radiopathomic features group (Supplementary Information SVI). The two steps mentioned above were utilized step by step as mitigation strategies for overfitting. Then, a few important features were identified for modeling. The strategies of early stop, regularization, and pruning were employed to further restrict overfitting risk during modeling.

**Fig. 1:**
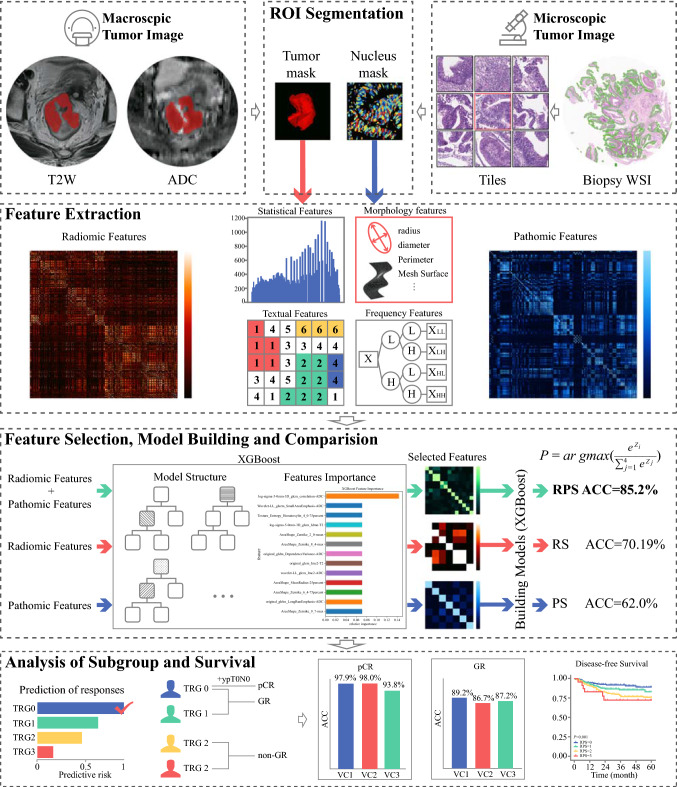
Flowchart of study. This study included ROI segmentation, feature extraction, feature selection, model training, signature construction, comparison, and analyses of subgroups and survival. Radiomic and pathomic features were extracted from mp-MRI or WSI of the same patient. The eXtreme Gradient Boosting (XGBoost) was used to select features and build models. Radiopathomic features were recorded after feature selection. Three signatures were constructed with different features by XGBoost, and model comparison was conducted to select the optimal model with the best performance for pretreatment prediction of TRG (i.e., discrepancies of pathological response). Subgroup and survival analyses based on RPS were used to evaluate the performance for pCR, GR, and survival prediction

For comparison, a radiomics signature (RS) was generated with features from mp-MRI alone, and another pathomics signature (PS) was generated with features from WSI alone. All models reached convergence conditions and were validated in the three external validation cohorts (VC1, VC2, and VC3). The flowchart of the study is shown in Fig. 1.

### Statistical Analysis

Quantitative statistics are presented as mean ± standard deviation (SD). Categorical variables were analyzed using *χ*^2^ or Fisher’s tests. ACC and Kappa coefficient were used to evaluate the overall performance of the multiple-category classification. Receiver operating characteristic (ROC) curves, area under curve (AUC), sensitivity, specificity, positive predictive value (PPV), and negative predictive value (NPV) were calculated for assessment of binary-category subgroup analysis. The bootstrap strategy (*N* = 1000) was applied to calculate the 95% confidence intervals (CIs). The net reclassification improvement (NRI) test was used for comparison among models at each category. The DeLong test of ROCs was used to evaluate improvement and overfiiting. For time-to-event endpoints, in addition to the Kaplan–Meier method, *P*-values were obtained from a stratified log-rank test, and the hazard ratio (HR) was calculated from a Cox proportional hazard model. The reported statistical significance levels were all two sided, with the statistical significance level set at 0.05. The statistical analyses were performed using the scikit-learn package (version 0.21.3) of Python (version 3.6.5) and R software (version 3.1.0).

## Results

### Clinical Characteristics

A total of 981 consecutive LARC patients (TRG 0, 24.65%, 240/981; TRG 1, 28.95%, 284/981; TRG 2, 41.90%, 411/981; TRG 3, 4.69%, 46/981; median age, 55 years) who received nCRT and TME radical surgery at the four hospitals were enrolled in this study. Proportion distributions of clinical characters to each TRG in the PC (SUSY6), VC1 (SYSUCC), VC2 (YNCH), and VC3 (PUCH) are presented in Supplementary Table S2. No significant differences were found in either baseline characters (*P* > 0.05) or the distribution of patients among different response (*P* > 0.05) between the PC and VCs.

### Prediction Performance of PRS and Comparison with RS and PS

**Fig. 2:**
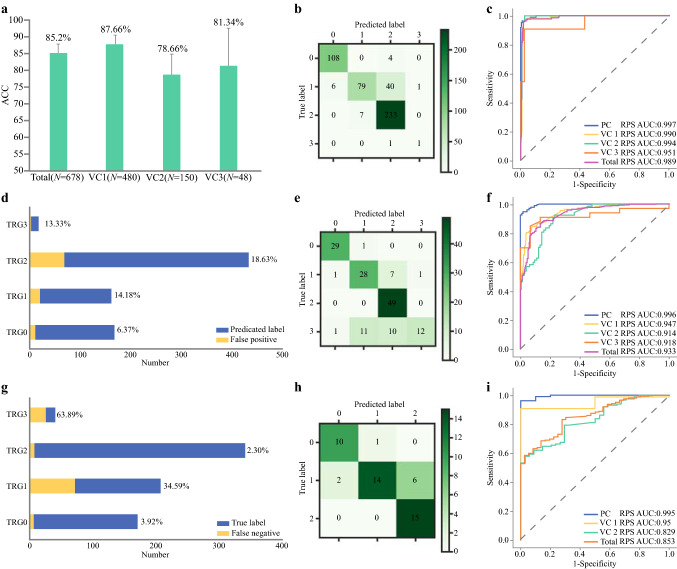
Overall performance of RPS: **a** accuracy of RPS in validation cohorts; **b**, **e**, **h** confusion matrixes of RPS in validation cohorts 1, 2, and 3. Row represents the true label, and column the predicted label; diagonal represents the number of patients whose predicted results were consistent with the true results; **c** receiver operating characteristic (ROC) curves of TRG0 versus TRG1–TRG3 in primary and validation cohorts; **c** ROC curves of ≤ TRG1 (TRG0–TRG1) versus TRG2–TRG3 in primary and validation cohorts; **c** ROC curves of ≤ TRG2 (TRG0–TRG2) versus TRG3 in primary and validation cohorts; **d** distribution of false-positive prediction at each grade in validation cohorts. Red indicates the number of false-positive samples in the prediction results, and blue indicates the total number of a certain type of prediction; **e** distribution of false-negative prediction at each grade in validation cohorts

A total of 96,796 images, including 96,076 MRI (T2WI and ADC) images and 981 biopsy H&E-stained slides digitalized images, were utilized for manual annotation of ROI and feature extraction. RPS was then constructed with radiopathomic features by XGBoost (Supplementary Information SVII). The overall performance of RPS among the different VCs was evaluated by ACC and Kappa coefficient according to the confusion matrix of prediction in VC1, VC2, and VC3. The ACC of RPS reached above or close to 80% in the VCs (Fig. 2a), being 12% and 25% higher than those of RS and PS, offering the highest ACC among VCs. The Kappa coefficient between RPS and true TRG was above 0.7 in all the VCs, being significantly better than for RS or PS (*P* < 0.01) (Table 1). Variance of ACC and Kappa value among validation cohorts was reduced to 3.46% (*P* > 0.05) and 0.04 (*P* > 0.05), respectively.

**Table 1:** Assessment of overall prediction performance of pathological responses

Metrics	Signatures	Total (*N* = 678)	VC1 (*N* = 480)	VC2 (*N* = 150)	VC3 (*N* = 48)
ACC (%) [95% CI]	RPS	85.2 [82.53–87.87]	87.66 [84.66–90.66]	78.66 [72.29–85.02]	81.34 [70.09–92.58]
	RS	70.19 [66.68–73.7]	75.51 [71.58–79.44]	62.08 [54.11–70.05]	41.9 [28.12–55.67]
	PS	62.0 [58.28–65.72]	62.67 [58.17–67.18]	60.56 [52.89–68.22]	58.29 [44.84–71.74]
Kappa coefficient [95% CI]	RPS	0.772 [0.733–0.812]	0.797 [0.75–0.845]	0.705 [0.619–0.791]	0.713 [0.549–0.878]
	RS	0.514 [0.466–0.563]	0.571 [0.508–0.635]	0.464 [0.371–0.558]	0.162 [0.006–0.318]
	PS	0.417 [0.36–0.473]	0.412 [0.345–0.48]	0.45 [0.354–0.545]	0.345 [0.126–0.564]

Statistical quantifications shown with 95% CI, when applicable

*VC1* validation cohort 1, *VC2* validation cohort 2, *VC3* validation cohort 3, *ACC* overall accuracy, *RPS* radiopathomics signature, *RS* radiomics signature, *PS* pathomics signature

The distinguishing ability of each level (TRG0, TRG1, TRG2, and TRG3) was evaluated by PPV and sensitivity. According to the precision and recall rate (Fig. 2b, c), RPS offered the best prediction for TRG0, with sensitivity and PPV above 90% in the validation cohorts (Table 2). The PPV of RPS dropped slightly for the prediction of TRG2, but with sensitivity exceeding 97%. Although the sensitivity and PPV for the prediction of TRG1 and TRG3 did not maintain superiority as for TRG0 or TRG1, the PPV still exceeded 80% in the validation cohorts. Compared with RS or PS, significant improvement was demonstrated by NRI (*P* < 0.01) (Table S4).

**Table 2:** Performance of radiopathomics signature at each category

Metric (%) [95% CI]	Total (*N* = 678)	VC1 (*N* = 480)	VC2 (*N* = 150)	VC3 (*N* = 48)
ACC	85.25 [82.55–87.96]	87.76 [84.86–90.66]	79.71 [72.52–84.9]	81.24 [70.15–92.34]
Sensitivity (TRG0)	96.08 [92.98–99.18]	96.49 [93.03–99.95]	96.62 [89.92–100.0]	91.3 [74.73–100.0]
Sensitivity (TRG1)	65.53 [58.62–72.44]	62.59 [54.22–70.95]	75.57 [61.75–89.4]	63.39 [42.96–83.82]
Sensitivity (TRG2)	97.67 [95.93–99.4]	97.16 [95.02–99.29]	100.0 [100.0–100.0]	100.0 [100.0 100.0]
Sensitivity (TRG3)	35.69 [20.0–51.38]	–	35.55 [19.99–51.12]	–
PPV (TRG0)	93.68 [89.93–97.43]	94.58 [90.48–98.69]	93.52 [84.68–100.0]	83.09 [61.19–100.0]
PPV (TRG1)	85.81 [80.03–91.59]	92.02 [86.19–97.85]	70.26 [56.42–84.1]	93.57 [81.1–100.0]
PPV (TRG2)	81.36 [77.32–85.39]	83.9 [79.6–88.19]	74.24 [63.57–84.92]	71.49 [52.04–90.94]
PPV (TRG3)	86.67 [69.29–100.0]	–	92.54 [78.21–100.0]	–

Statistical quantifications shown with 95% CI, when applicable

*TRG* tumor regression grade, *VC1* validation cohort 1, *VC2* validation cohort 2, *VC3* validation cohort 3, *PPV* positive predictive value, ‘–’ insufficient sample distribution for evaluation

**Fig. 3:**
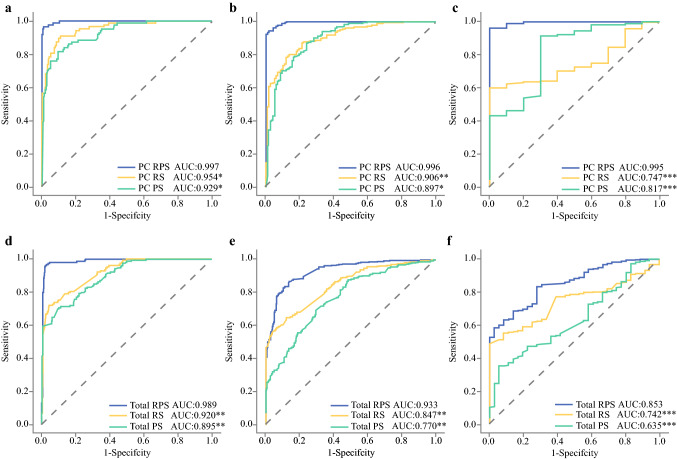
Comparison of receiver operating characteristic (ROC) curves among different signatures in primary cohort and patients in all validation cohorts: **a**, **d** TRG0 (TRG0) versus TRG1–TRG3 in primary and validation cohorts; **b**, **e** ≤ TRG1 (TRG0–TRG1) versus TRG1–TRG3 in primary and validation cohorts; **c**, **f** ≤ TRG2 (TRG0–TRG2) versus TRG3 in primary and validation cohorts. AUCs of RPS were statistically compared with AUC of RS and PS (*P < 0.05; **P < 0.01; ***P < 0.001)

RPS was also grouped for classification of TRG0 (TRG0), ≤ TRG1 (TRG0 and TRG1), and ≤ TRG2 (TRG0, TRG1, and TRG2), and AUCs exceeded 0.95, 0.90, and 0.80, respectively, for stratifications in the PC and VCs (Table S3; Fig. 1c, f, i). However, the AUCs dropped for ≤ TRG2 in VC3 and the collection of all patients in the validation cohorts because of the limited number of negative samples (TRG3), albeit still showing sensitivity above or close to 95% and PPV above 80%. Then, ROC curves and AUCs were utilized for comparison of RPS versus RS and PS. RPS for TRG0 yielded the highest AUCs in the PC and VCs. Compared with AUCs in the PC, AUCs of RPS dropped for ≤ TRG1 and ≤ TRG2 in the validation cohorts, albeit still significantly higher than those of RS or PS (*P* < 0.01) (Fig. 3). From a DeLong test of ROC curves and AUCs, RPS for TRG0, ≤ TRG1, and ≤ TRG2 revealed both significantly incremental performance (*P* < 0.05) and no statistical difference between the different centers (*P* > 0.05).

### Subgroup Analyses Based on Radiopathomics Signature

For further analysis, RPS was divided into two subgroups, viz. pCR (TRG0 and ypT0N0) and GR (TRG0 and TRG1), to correspond to clinical use. All the predicted results were validated at the different centers (Supplementary Table S4).

The pCR subgroup yielded ACC of 97.65% (95% CI, 96.5–98.8%) for the total validation patients, and the ACCs of all the validation cohorts were larger than or close to 95%. The sensitivity, specificity, PPV, and NPV were all above or close to 95% in VC1 and VC2.

The subgroup of GR yielded an ACC of 88.47% (95% CI, 86.07–90.87%) for the total validation patients, and the ACCs of all validation cohorts were larger than or close to 87%. The specificity and PPV in VC1 and VC3 were above 96%, with sensitivity above 80%.

### Prognosis Based on RPS

**Fig. 4:**
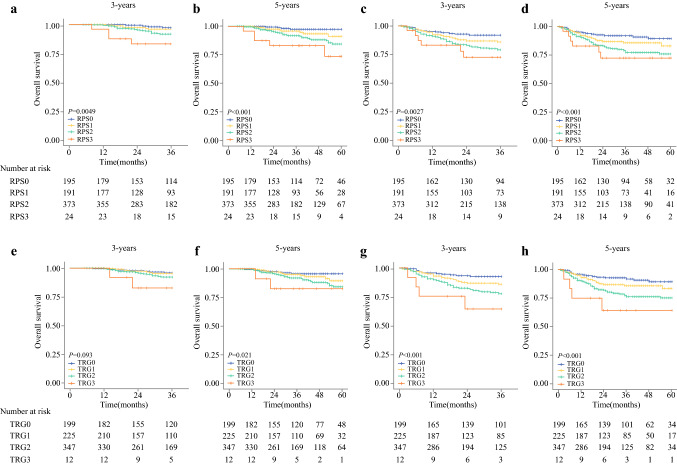
Kaplan–Meier survival curves: **a**, **b** overall survival curves at 3 or 5 years based on RPS; **c**, **d** disease-free survival curves at 3 or 5 years based on RPS; **e**, **f** overall survival curves at 3 or 5 years based on true four-level pathological response; **g**, **h** disease-free survival curves at 3 or 5 years based on true four-level pathological response

The Kaplan–Meier curve analysis showed that each RPS was related to distinct probabilities of overall survival (OS) and disease-free survival (DFS) for LARC patients (all *P* < 0.01) (Fig. 4). With increasing TRG level, both OS and DFS decreased at both 3 and 5 years, while TRG0 and TRG3 showed the best and worst survival outcomes, respectively. Moreover, multivariate Cox regression analysis (with true TRG and Rp-Grade) was used to assess whether RPS was independent of true TRG in predicting survival and confirmed that RPS was an independent prognostic factor for OS (HR 6.975, 95% CI 4.191–13.12, *P* = 0.003) and DFS (HR 3.831, 95% CI 2.204–4.917, *P* = 0.007).

## Discussion

In clinical practice, it is very difficult to rely solely on radiographic or clinical diagnostic information to obtain patients’ different levels of pathological response for treatment optimization prior to nCRT. How to integrate lesion information on a more visual scale to develop a more reliable and generalized method to predict different responses remains a challenging issue.

In this multicenter prospective study, the accuracy of mp-MRI alone (RS), WSI alone (PS), and combining both mp-MRI and WSI (RPS) in pretreatment prediction of TRG (i.e., pathological response at different levels) was compared. We demonstrated outperformance and significant improvement for RPS, which was generated by the radipatomics strategy combining both radiological information of whole tumor (mp-MRI) and pathological information of local lesions (WSI). RPS achieved the highest overall performance among the three signatures, yielding ACC of 78.66–87.76% versus 41.9–75.51% (RS) and 58.29–62.0% (PS). Kappa coefficients of RPS lay in the range of 0.6–0.8, indicating substantial agreement with the reference surgical specimen,^25^ and was also higher than RS or PS.

Furthermore, signatures were grouped for classifying TRG0 (TRG0), ≤ TRG1 (TRG0 and TRG1), and ≤ TRG2 (TRG0, TRG1, and TRG2). AUCs of RPS reached over 0.95, 0.90, and 0.80 for stratifications in the PC and VCs, respectively. The higher sensitivity and PPV in TRG0 and ≤ TRG1 confirmed the ability of the model to discriminate patients who could achieve greater benefit from standard nCRT. The higher sensitivity and PPV of RPS in ≤ TRG2 could decrease the rate of missing patients who were suitable for nCRT. Comparison between signatures based on the group classification was assessed by ROC curves and DeLong test and verified the significant advantages of RPS in the PC (*P* < 0.05) and VCs (*P* < 0.01) again.

Although some categories of TRG revealed distinct probabilities of LARC patients’ survival, especially TRG3, the differences between the Kaplan–Meier curves (Fig. 4) suggested that it was difficult for the treatments to be graded among all four TRGs. Therefore, according to the guideline,^26^ the RPS combining posttreatment pathological evaluation of lymph nodes was grouped for predicting pCR status pretreatment (ACC of 97.65%), which even exceeded the accuracy of 94.08% for 222 patients achieved by pCR evaluation using posttreatment mp-MRI.^15^ The GR subgroup (TRG0 and TRG1) also yielded satisfactory prediction accuracy (ACC of 88.47%) in the VCs. Prediction of pCR or GR can reliably assist doctors in accurately identifying patients with pCR for whom a “wait and see” approach,^15^ local excision,^27^ or weighting benefits of neoadjuvant therapy against drug toxicity^28^ may be most appropriate. More importantly, each category of RPS revealed distinct probabilities of LARC patients’ OS and DFS (*P* < 0.05) and was a prognosis factor independent of true TRG (*P* < 0.01). Combined with clinical TNM stage, this may be able to optimize the existing standard for the definition of patients who rely on nCRT.^5^

The first finding of this study is that the prediction model using a radiomics strategy could associate mp-MRI with different levels of response to nCRT. Radiomics analysis integrating many high-dimensional imaging features can be used to predict neoadjuvant therapy effects that are difficult to detect visually and may perform relatively well before treatment. Quantitative and high-dimensional features from mp-MRI were demonstrated to qualitatively predict pCR and GR^17^ and to enhance the limited accuracy of models by combining information from more imaging modalities.^18^ RS, which was constructed with mp-MRI alone, yielded overall ACC of 75.51% in the largest VC (VC1, *n* = 480), and achieved AUCs of 0.92 (TRG0), 0.85 (≤ TRG1), and 0.74 (≤ TRG2) in the other VCs. There was no obvious overfitting between the PC and VCs.

The second finding is that quantitative tumor pathology information from WSI is useful to enrich lesion descriptors and is related to the response to nCRT. Microscopic pathology image features have been explored to screen abnormality and detect disease,^15^ classify molecular subtype,^29^,^30^ distinguish susceptive responders^29^,^30^ or intractable patients,^31^ as well as predict survival outcomes.^32^,^33^ However, it is unfortunate that pathological information has not been mined from biopsy specimens obtained from colonoscopy to predict tumor response, given that biopsy is an essential examination for LARC patients.^34^ Levels of tumor cell differentiation^32^ were one highlight among numerous pathological descriptors, which could be calculated by automated histopathological imaging analysis systems using WSI. With this method, extensive, quantitative, and high-dimensional tumor microinformation was obtained, also showing potential to integrate with other types of features to build a multiomics prediction model for prognosis.^22^ During our experiments, we utilized the same strategy to extract nucleus features from WSI (named pathomic features) and generated PS. The overall ACC of PS was above or close to 60% in the VCs. The AUCs of PS in TRG0, ≤ TRG1, and ≤ TRG2 were 0.895, 0.77, and 0.635 in the VCs, respectively. Compared with RS, the AUC of PS in TRG0 was close to R-Grade but significantly decreased in ≤ TRG1 and ≤ TRG2 (*P* < 0.05) in the VCs. PS and pathomic features showed relevance for different response, but PS slightly lacked sufficient reliability as an independent signature for response prediction.

The most important finding of this work is the radiopathomics strategy, which strengthens the radiomics strategy by adding pathological knowledge. This strategy mines complementary information provided by WSI and mp-MRI and enriches the descriptors of tumors to predict discrepancies in the pathological response to nCRT. Highly correlated with pathological response, the set of selected features with limited redundant information was the most important effect reducing modeling overfitting. The power of the radiopathomics strategy achieved a significant improvement compared with the signatures constructed using mp-MRI or WSI alone (*P* < 0.05). According to radiology, the mp-MRI contained phenotype (T2) and microcirculation (ADC) heterogeneous information for the whole tumor.^33^ According to pathology, biopsy WSI reflected pathological and gene-related information through nucleus features at a microscopic level.^35^ There was correlation between the information between the different observation scales, although most were independent from each other. In our study, 1404 features from MRI and 770 features from biopsy WSI of each patient were extracted. Heat maps of feature analysis with Pearson coefficient showed little correlation between radiomic and pathomic features (Supplementary Fig. S4), indicating that there was not too much redundant information between radiomic and pathomic features in our feature extraction. Then, the radiopathomics strategy found more interactive features with higher Spearman correlation coefficient of tumor response (Supplementary Fig. S5), indicating that the selected features were more relevant to response at different levels, and their combination was more powerful. The selected features reflected the shape and texture features of the tumor in MRI, and the shape or texture information of the nucleus in biopsy WSI (Supplementary Table S5). Radiopathomics integrating these features provides a more comprehensive overall picture of the tumor. Hence, RPS showed satisfactorily incremental performance without overfitting according to this multicenter validation. The subgroup of RPS was also higher than in previous research^17^,^18^,^36^ because of the use of the radiopathomics strategy and a state-of-the-art modeling method (XGBoost). XGBoost is a recent method that has offered a breakthrough in the field of data mining.^37^ The second reason for restricting overfitting was that XGBoost obtained more diverse optimization strategies in weight updating, loss function, regularization, and pruning. Last but not least, samples with a sufficient number and high quality in the training phase were also important for reducing overfitting. Consequently, our proposed radiopathomics strategy and signature avoid the limitation of only using medical imaging to depict the whole tumor and decrease the potential risk of overlooking tumor heterogeneity by adding microscale pathological information. A more accurate signature without overfitting for pretreatment prediction of different degrees of efficacy of nCRT based on routine inspection without additional examination, combining features of medical and digital pathology imaging modalities, provided greater reliability for better and more personalized treatment planning for patients with LARC who underwent nCRT.

In conclusion, the RPS proposed herein based on a radiopathomics strategy using pretreatment mp-MRI and WSI from H&E-stained biopsy specimens together, thereby combining radiological information of the whole tumor and pathological information of local lesions, provides a new approach for pretreatment prediction of discrepancies of pathological response to restage LARC patients who undergo nCRT. RPS could be useful in individualized clinical decision-making by providing radiologists and oncologists with a potential tool for more detailed response prediction with hierarchical prognostic relevance.

## Electronic supplementary material

Below is the link to the electronic supplementary material.

Supplementary material 1 (DOCX 95739 kb)

Supplementary material 2 (DOCX 39 kb)
