# Supplementary material for: Multiparametric MRI and Whole Slide Image-Based Pretreatment Prediction of Pathological Response to Neoadjuvant Chemoradiotherapy in Rectal Cancer: A Multicenter Radiopathomic Study
Source: Ann Surg Oncol. 2020 Jul 29;27(11):4296–306. doi: 10.1245/s10434-020-08659-4 (PMC7497677; doi:10.1245/s10434-020-08659-4)
Supplement: Supplementary file 1 — Supplementary material 1 (DOCX 95739 kb) [file 10434_2020_8659_MOESM1_ESM.docx]

**SUPPLEMENTARY IMAGES AND TABLES**

1. **Figure S1**
2. **Figure S2**
3. **Figure S3**
4. **Figure S4**
5. **Figure S5**
6. **Table S1**
7. **Table S2**
8. **Table S3**
9. **Table S4**
10. **Table S5**
11. **Table S6**

|  |
| --- |

**Figure S1.** Patient recruitment and study design. In total, 1072 of 981 patients with pretreatment multiparametric MRI (mp-MRI) and whole slide image (WSI) from four Chinese hospitals were enrolled in this study for model construction and validation.

| 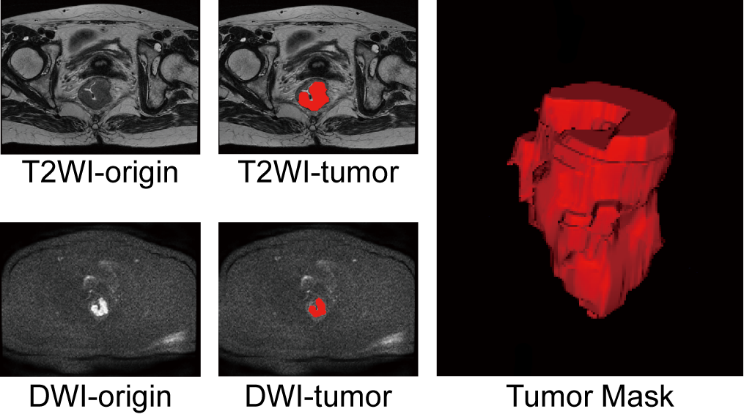 |
| --- |

**Figure S2.** Annotation of multiparametric MRI.

| **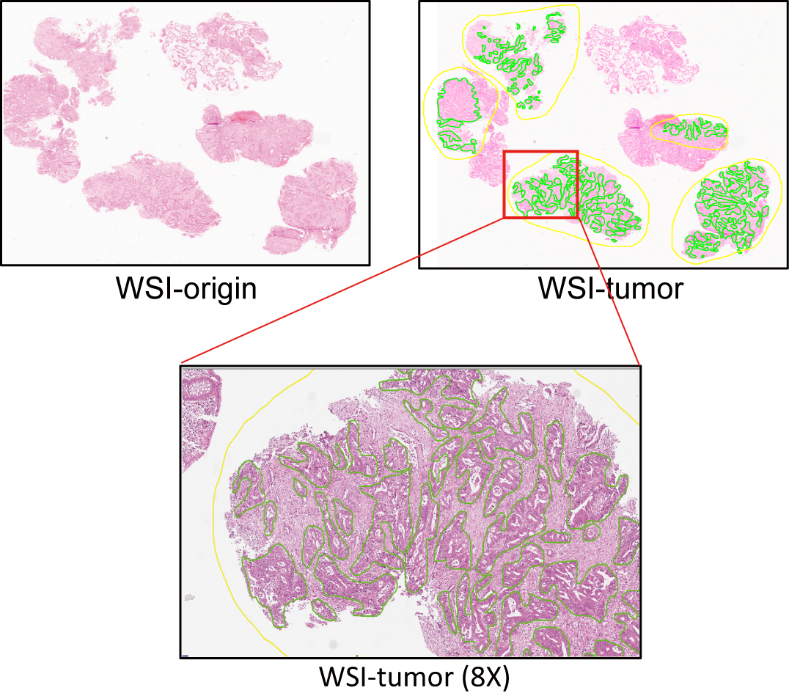** |
| --- |

**Figure S3.** Annotation of whole slide image.

| **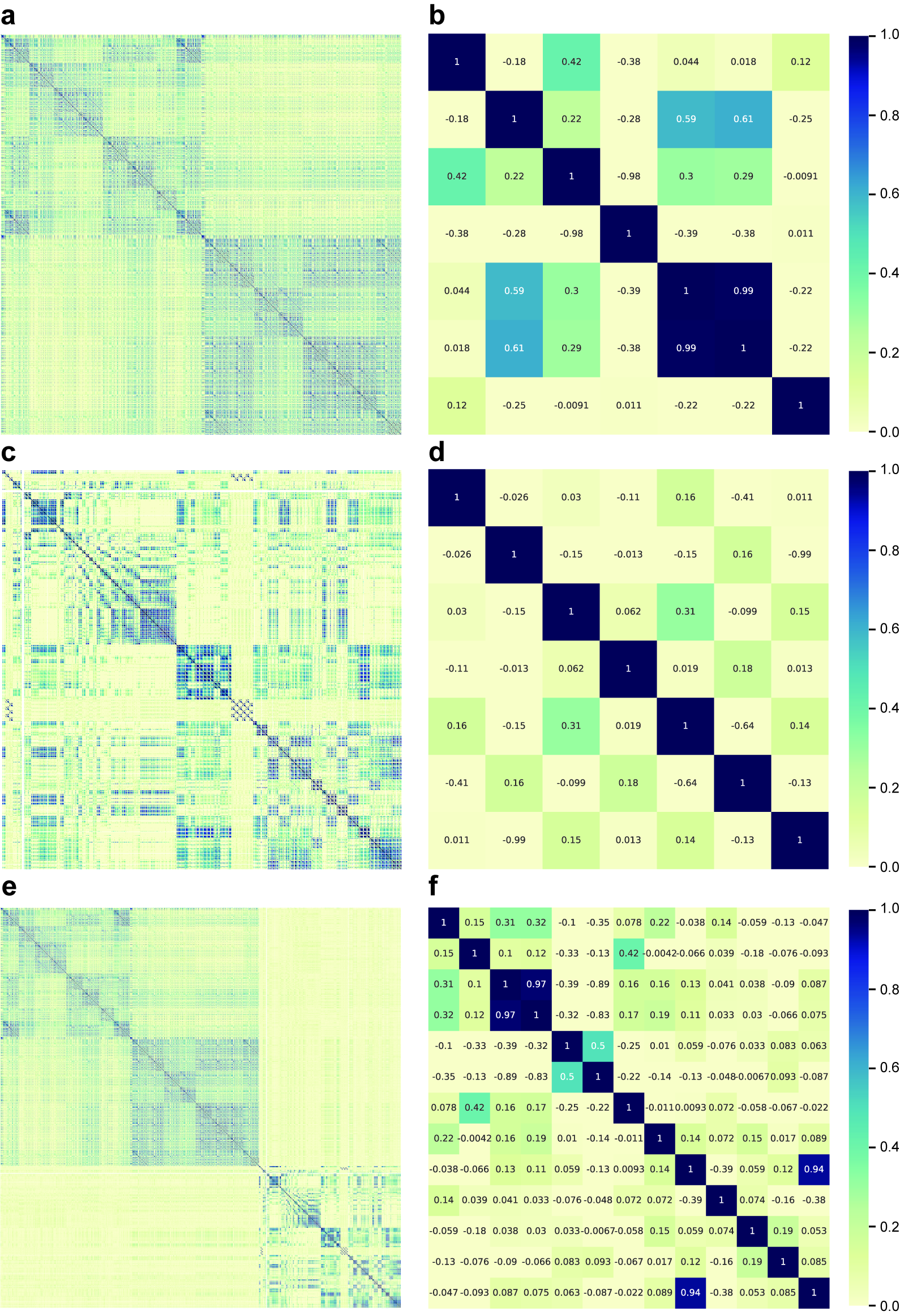** |
| --- |

**Figure S4.** Feature correlation heat map. (a,b) Original features correlation heat map of total radiomic features and heat map after screening. (c,d) Original feature heat map of total pathomics features and heat map after screening. (e) Original features’ heat map of combination of radiomic features and pathomic features. (f) Correlation heat map of radiopathomic features. Digital identifications in the maps were Pearson coefficients.

| 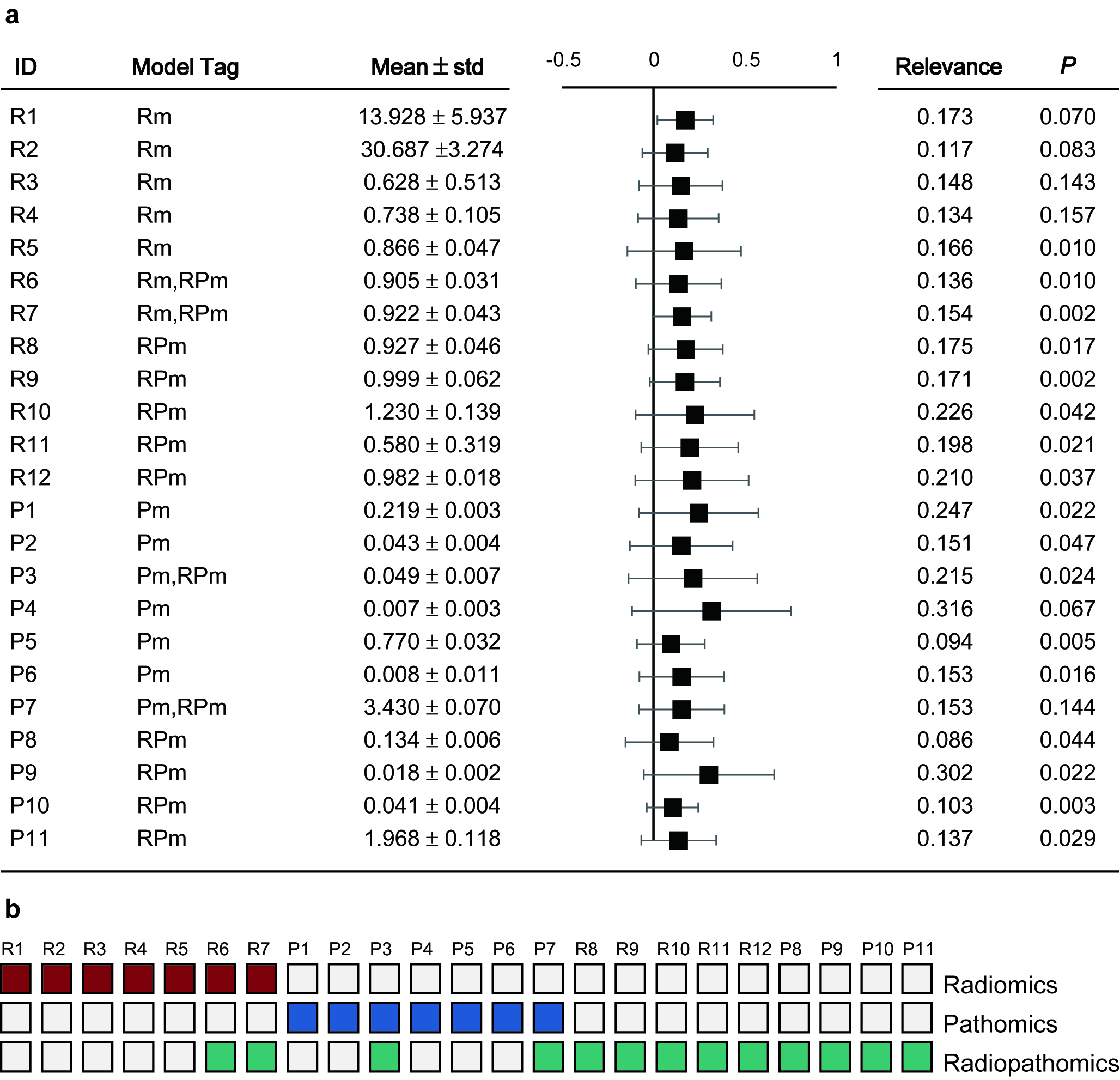 |
| --- |

**Figure S5.** Relevance of selected features of models. (a) Forest plots of Spearman correlation between features and real 4-level pathological response. ID, consists of feature type and feature number; R, radiomic features from MRI; P, pathomic features from whole slide image; Model Tag, identification of the models; Rm, radiomics model; Pm, pathomics model; RPm, radiopathomics model; Mean±std, mean of the features; Relevance, Spearman coefficient; (b) Difference of models according modeling features.

**Table S1.** The MR image acquisition parameters of the four centers

| **Hospital** | **Scanner** | **Patients No.** | **Sequence** | **TR/TE**  **(ms)** | **FOV**  **(mm)** | **Matrix** | **Slice Thickness (mm)** | **Slice Gap**  **(mm)** | **Slices** | **Flip Angle** |
| --- | --- | --- | --- | --- | --- | --- | --- | --- | --- | --- |
| the Sixth Affiliated Hospital of Sun Yat-sen University | GE 3.0T  (OPTIMA) | 303 | T2WI | 4300/104 | 100×100 | 288×256 | 3 | 6 | 26 | 90° |
|  |  |  | DWI | 4500/92 | 100×100 | 192×192 | 3 | 6 | 20 | 90° |
| Sun Yat-sen University Cancer Center | Philips 3.0T  (Achieva) | 45 | T2WI | 2852.318/90 | 150 | 516×510 | 5 | 6 | 32 | 90° |
|  |  |  | DWI | 2374.324/51.634 | 70 | 132×127 | 5 | 5.5 | 36 | 90° |
|  | GE 3.0T  (DISCOVERY) | 105 | T2WI | 6480.728/81.692 | 100 | 384×384 | 5 | 6 | 36 | 90° |
|  |  |  | DWI | 4000/56.7 | 80 | 128×128 | 5 | 6 | 24 | 90° |
|  | GE 1.5T  (SIGNA) | 91 | T2WI | 6000/92.184 | 60 | 320×224 | 8 | 10 | 24 | 90° |
|  |  |  | DWI | 5000/75.6 | 100 | 128×128 | 6 | 7 | 25 | 90° |
|  | SIEMENS 3.0T  (TrioTim) | 239 | T2WI | 3000/84 | 75 | 384×230 | 5 | 6 | 30 | 90° |
|  |  |  | DWI | 4000/70 | 50 | 128×64 | 5 | 6 | 24 | 90° |
| Yunnan Cancer Hospital | Siemens 1.5T  (Avanto) | 80 | T2WI | 3200/100 | 200×200 | 288×320 | 4 | 0.4 | 20 | 90° |
|  |  |  | DWI | 4900/84 | 185×340 | 220×220 | 4 | 0.8 | 24 | 90° |
|  | Philip 3.0T  (Ingenia) | 70 | T2WI | 2100/100 | 200×200 | 400×284 | 4 | 0.4 | 18 | 90° |
|  |  |  | DWI | 3070/62 | 280×342 | 108×129 | 3 | 0.6 | 40 | 90° |
| Peking University Cancer Hospital | GE1.5T  (SIGNA) | 32 | T2WI | 3400/90 | 260×320 | 348×299 | 3 | 0.3 | 44 | 120° |
|  |  |  | DWI | 2000/103 | 320×320 | 160×160 | 5 | 1 | 32 | 90° |
|  | GE 3.0T  (DISCOVERY) | 16 | T2WI | 3600/54 | 340×319 | 384×384 | 4 | 0.4 | 28 | 120° |
|  |  |  | DWI | 6700/93 | 340×153 | 200×200 | 4 | 0.4 | 28 | 120° |

Note: FOV, field of view; TR, repetition time; TE, echo time; T2WI, T2 weighted imaging; DWI, diffusion weighted imaging.

**Table S2.** Clinical characteristics

| Characteristics | PC (*N*=303) | | | | *P* | VC1 (*N*=480) | | | | *P* | VC2 (*N* =150) | | | | *P* | VC3 (*N* =48) | | | | ***P*** |
| --- | --- | --- | --- | --- | --- | --- | --- | --- | --- | --- | --- | --- | --- | --- | --- | --- | --- | --- | --- | --- |
|  | TRG0 (%) | TRG1 (%) | TRG2 (%) | TRG3 (%) |  | TRG0 (%) | TRG1  (%) | TRG2  (%) | TRG3  (%) |  | TRG0 (%) | TRG1 (%) | TRG2 (%) | TRG3 (%) |  | TRG0 (%) | TRG1  (%) | TRG2  (%) | TRG3 (%) |  |
|  | (*N*=87) | (*N* =99) | (*N* =107) | (*N* =10) |  | (*N* =112) | (*N* =126) | (*N* =240) | (*N* =2) |  | (*N* =30) | (*N* =37) | (*N* =49) | (*N* =34) |  | (*N* =11) | (*N* =22) | (*N* =15) | (*N* =0) |  |
| Age |  |  |  |  | 0.29 |  |  |  |  | 0.546 |  |  |  |  | 0.658 |  |  |  |  | 0.295 |
| ≤ 55 | 48  (31.8) | 48  (31.8) | 48  (31.8) | 7  (4.6) |  | 55  (24.0) | 62  (27.1) | 112  (48.9) | 0  (0) |  | 15  (23.1) | 16  (24.6) | 18  (27.7) | 16  (24.6) |  | 8  (32.0) | 10  (40.0) | 7  (28.0) | 0  (0) |  |
| > 55 | 39  (25.7) | 51  (33.6) | 59  (38.8) | 3  (2.0) |  | 57  (22.7) | 64  (25.5) | 128  (51.0) | 2  (0.8) |  | 15  (17.6) | 21  (24.7) | 31  (36.5) | 18  (21.2) |  | 3  (12.0) | 12  (48.0) | 8  (32.0) | 0  (0) |  |
| Sex |  |  |  |  | 0.142 |  |  |  |  | 0.823 |  |  |  |  | 0.056 |  |  |  |  | 0.467 |
| Male | 62  (29.0) | 65  (30.4) | 77  (36.0) | 10  (4.7) |  | 75  (23.7) | 86  (27.2) | 154  (48.7) | 1  (0.3) |  | 14 (14.0) | 27 (27.0) | 33 (33.0) | 26  (26.0) |  | 5  (26.3) | 10  (52.6) | 4  (21.1) | 0  (0) |  |
| Female | 25  (28.1) | 34  (38.2) | 30  (33.7) | 0  (0) |  | 37  (22.6) | 40  (24.4) | 86  (52.4) | 1  (0.6) |  | 16 (32.0) | 10 (20.0) | 16 (32.0) | 8  (16.0) |  | 6  (31.6) | 12  (63.2) | 11  (57.9) | 0  (0) |  |
| Clinical T stage |  |  |  |  | 0.147 |  |  |  |  | 0.99 |  |  |  |  | 0.124 |  |  |  |  | 0.716 |
| cT1 | 0  (0) | 0  (0) | 0  (0) | 0  (0) |  | 1  (50.0) | 0  (0) | 1  (50.0) | 0  (0) |  | 0  (0) | 0  (0) | 0  (0) | 0  (0) |  | 0  (0) | 0  (0) | 0  (0) | 0  (0) |  |
| cT2 | 7  (63.6) | 0  (0) | 4  (36.4) | 0  (0) |  | 4  (23.5) | 4  (23.5) | 9  (52.9) | 0  (0) |  | 2  (66.7) | 0  (0) | 1  (33.3) | 0  (0) |  | 3  (33.3) | 4  (44.4) | 2  (22.2) | 0  (0) |  |
| cT3 | 61  (27.4) | 80  (35.9) | 75  (33.6) | 7  (3.1) |  | 69  (23.6) | 80  (27.4) | 141  (48.3) | 2  (0.7) |  | 14  (23.7) | 10  (16.9) | 17  (28.8) | 18  (30.5) |  | 8  (88.9) | 17  (188.9) | 12  (133.3) | 0  (0) |  |
| cT4a | 9  (25.7) | 8  (22.9) | 17  (48.6) | 1  (2.9) |  | 32  (22.2) | 37  (25.7) | 75  (52.1) | 0  (0) |  | 13  (15.1) | 26  (30.2) | 31  (36.0) | 16  (18.6) |  | 0  (0) | 0  (0) | 0  (0) | 0  (0) |  |
| cT4b | 10  (29.4) | 11  (32.4) | 11  (32.4) | 2  (5.9) |  | 6  (24.0) | 5  (20.0) | 14  (56.0) | 0  (0) |  | 1  (50.0) | 1  (50.0) | 0  (0) | 0  (0) |  | 0  (0) | 0  (0) | 0  (0) | 0  (0) |  |
| Clinical N stage |  |  |  |  | 0.094 |  |  |  |  | 0.517 |  |  |  |  | 0.024* |  |  |  |  | 0.268 |
| cN0 | 15  (28.8) | 15  (28.8) | 21  (40.4) | 1  (1.9) |  | 24  (30.8) | 21  (26.9) | 33  (42.3) | 0  (0) |  | 1  (3.3) | 5  (16.7) | 12  (40.0) | 12  (40.0) |  | 1  (12.5) | 3  (37.5) | 4  (50.0) | 0  (0) |  |
| cN1 | 39  (31.7) | 31 (25.2) | 46  (37.4) | 7  (5.7) |  | 59  (22.3) | 67  (25.3) | 137  (51.7) | 2  (0.8) |  | 22  (24.2) | 27  (29.7) | 28  (30.8) | 14  (15.4) |  | 4  (40.0) | 2  (20.0) | 4  (40.0) | 0  (0) |  |
| cN2 | 33  (25.8) | 53  (41.4) | 40  (31.3) | 2  (1.6) |  | 29  (21.2) | 38  (27.7) | 70  (51.1) | 0  (0) |  | 7  (24.1) | 5  (17.2) | 9  (31.0) | 8  (27.6) |  | 2  (9.5) | 5  (23.8) | 14  (66.7) | 0  (0) |  |
| Tumor differentiation |  |  |  |  | 0.362 |  |  |  |  | 0.036* |  |  |  |  | 0.747 |  |  |  |  | 0.928 |
| High | 20  (23.0) | 32  (36.8) | 33  (37.9) | 2  (2.3) |  | 4  (44.4) | 4  (44.4) | 1  (11.1) | 0  (0) |  | 1  (50.0) | 0  (0) | 1  (50.0) | 0  (0) |  | 0  (0) | 0  (0) | 0  (0) | 0  (0) |  |
| Median | 58  (29.3) | 63  (31.8) | 70  (35.4) | 7  (3.5) |  | 88  (22.3) | 94  (23.9) | 210  (53.3) | 2  (0.5) |  | 28  (20.4) | 34  (24.8) | 43  (31.4) | 32  (23.4) |  | 9  (22.0) | 19  (46.3) | 13  (31.7) | 0  (0) |  |
| Low | 9  (50.0) | 4  (22.2) | 4  (22.2) | 1  (5.6) |  | 20  (26.0) | 28  (36.4) | 29  (37.7) | 0  (0) |  | 1  (9.1) | 3  (27.3) | 5  (45.5) | 2  (18.2) |  | 2  (4.9) | 3  (7.3) | 2  (4.9) | 0  (0) |  |

Note: Qualitative variables are in n (%), and quantitative variables are in mean±SD, when appropriate. Chi-square or Fisher’s exact tests, as appropriate, were used to compare the differences in categorical variables. * P <0.05.

VC1, validation cohort1; VC2, validation cohort2; VC3, validation cohort3.

**Table S3.** Performance of radiopathomics signature for predicting responses in primary and validation cohorts

|  |  | N(P) | AUC | Sensitivity (%) | Specificity (%) | PPV (%) | NPV (%) |
| --- | --- | --- | --- | --- | --- | --- | --- |
| TRG0 | PC | 303 (28.7%) | 0.99  [0.99-1.00] | 94.2  [89.3-99.0] | 99.6  [98.8-100.0] | 99.0  [96.9-100.0] | 97.6  [95.7-99.6] |
|  | Total | 678 (22.6%) | 0.98  [0.98-0.99] | 96.4  [93.6-99.3] | 97.1  [95.6-98.5] | 90.7  [86.3-95.2] | 98.9  [98.0-99.8] |
|  | VC1 | 480 (23.3%) | 0.98  [0.98-0.99] | 96.4  [93.3-99.5] | 97.6  [96.2-98.9] | 92.2  [87.9-96.6] | 98.9  [97.9-99.8] |
|  | VC2 | 150 (20.0%) | 0.99  [0.98-1.00] | 100.0  [100.0-100.0] | 96.6  [93.3-99.9] | 88.5  [82.2-94.9] | 100.0  [100.0-100.0] |
|  | VC3 | 48 (22.9%) | 0.94  [0.86-1.00] | 89.7  [70.3-100.0] | 94.3  [87.2-100.0] | 81.3  [74.6-88.0] | 97.2  [92.5-100.0] |
| ≤TRG1 | PC | 303 (61.4%) | 0.99  [0.99-1.00] | 94.8  [91.1-98.5] | 95.7  [92.3-99.2] | 97.3  [95.2-99.4] | 92.0  [86.4-97.6] |
|  | Total | 678 (49.9%) | 0.93  [0.91-0.95] | 80.3  [76.1-84.6] | 91.6  [88.4-94.8] | 90.6  [87.1-94.1] | 82.2  [77.8-86.7] |
|  | VC1 | 480 (49.6%) | 0.94  [0.92-0.96] | 80.5  [75.6-85.3] | 94.6  [91.8-97.3] | 93.6  [90.6-96.6] | 83.1  [78.5-87.5] |
|  | VC2 | 150 (44.7%) | 0.91  [0.87-0.95] | 83.58  [74.9-92.2] | 82.3  [74.6-89.9] | 78.7  [69.6-87.8] | 86.4  [78.6-94.1] |
|  | VC3 | 48 (68.8%) | 0.91  [0.84-0.99] | 70.8  [60.2-81.5] | 94.1  [85.4-100.0] | 96.2  [88.5-100.0] | 59.8  [40.2-79.4] |
| ≤TRG2 | PC | 303 (96.7%) | 0.99  [0.98-1.00] | 96.8  [94.9-98.6] | 90.7  [70.0-100.0] | 99.7  [99.0-100.0] | 47.1  [22.5-71.8] |
|  | Total | 678 (94.7%) | 0.849  [0.798-0.901] | 95.7  [94.2-97.2] | 32.4  [16.5-48.3] | 96.2  [94.7-97.7] | 29.7  [16.5-42.89] |
|  | VC1 | 480 (99.6%) | 0.95  [0.88-1.00] | 99.7  [99.2-100.0] | 100.0  [100.0-100.0] | 99.7  [99.3-100.0] | 5.6  [0-16.1] |
|  | VC2 | 150 (77.3%) | 0.82  [0.75-0.89] | 94.9  [91.3-98.5] | 31.5  [17.1-45.9] | 82.6  [76.8-88.6] | 64.5  [42.9-86.1] |
|  | VC3 | 48 (100.0%) | - | 87.1  [76.9-97.1] | 100.0  [100.0-100.0] | 100.0  [100.0-100.0] | 100.0  [100.0-100.0] |

Note: Statistical quantifications were demonstrated with 95% CI, when applicable.

AUC, area under the receiver operating characteristic curve; N, number of patients; P, prevalence; NPV, negative predictive value; PPV, positive predictive value; PC, primary cohort; VC1, validation cohort1; VC2, validation cohort2; VC3, validation cohort3; Total, collection of patients in all validation cohorts; TRG0, tumor regression grade is equal to level 0; ≤TRG1, collection of TRG0 and TRG1; ≤TRG2, collection of TRG0,TRG1 and TRG2. '-', insufficient samples distribution for evaluation.

**Table S4.** Incremental performance of RPS compared with RS or PS in validation cohorts

| TRG | Signatures | VC1 (*N*=481) | | VC2 (*N*=150) | | VC3 (*N*=48) | |
| --- | --- | --- | --- | --- | --- | --- | --- |
|  |  | NRI  [95% CI] | *P* | NRI  [95% CI] | *P* | NRI  [95% CI] | *P* |
| TRG0 | RS | 0.153  [0.080-0.226] | <0.001 | 0.0917  [0.011-0.174] | 0.021 | 0.584  [0.413-0.755] | <0.001 |
|  | PS | 0.373  [0.276-0.471] | <0.001 | 0.116  [0.049-0.183] | 0.0116 | 0.479  [0.322-0.636] | 0.005 |
| TRG1 | RS | 0.170  [0.079-0.259] | <0.001 | 0.243  [0.068-0.419] | 0.007 | 0.409  [0.256-0.572] | 0.006 |
|  | PS | 0.323  [0.221-0.425] | <0.001 | 0.333  [0.152-0.515] | <0.001 | 0.276  [0.151-0.427] | 0.046 |
| TRG2 | RS | 0.116  [0.0597-0.173] | <0.001 | 0.238  [0.150-0.325] | <0.001 | 0.468  [0.193-0.744] | <0.001 |
|  | PS | 0.375  [0.295-0.455] | <0.001 | 0.268  [0.175-0.361] | <0.001 | 0.344  [0.144-0.544] | 0.011 |
| TRG3 | RS | - | - | 0.324  [0.165-0.483] | <0.001 | - | - |
|  | PS | - | - | 0.315  [0.157-0.473] | <0.001 | - | - |

Note: Statistical quantifications were demonstrated with 95% CI, when applicable.

TRG, tumor regression grade; VC1, validation cohort1; VC2, validation cohort2; VC3, validation cohort3; NRI, Net Reclassification Improvement; RS, radiomics signature; PS, pathomics signature; '-', insufficient samples distribution for evaluation.

**Table S5.** Subgroup analyses of pCR and GR

| Subgroups | Metrics | Total | VC1 | VC2 | VC3 |
| --- | --- | --- | --- | --- | --- |
| pCR | ACC (%)  [95% CI] | 97.65  [96.5-98.8] | 97.88  [96.6-99.16] | 97.98  [95.67 100.0] | 93.84  [87.19-98.48] |
|  | Sensitivity (%)  [95% CI] | 96.14  [93.09-99.2] | 96.4  [92.9-99.91] | 96.72  [90.24 100.0] | 90.89  [73.83-100.0] |
|  | Specificity (%)  [95% CI] | 98.08  [96.9-99.27] | 98.33  [96.99-99.67] | 98.3  [95.98 100.0] | 94.76  [87.56-100.0] |
|  | PPV (%)  [95% CI] | 93.58  [89.66-97.49] | 94.58  [90.29-98.87] | 93.42  [84.45-100.0] | 83.66  [61.59-100.0] |
|  | NPV (%)  [95% CI] | 98.87  [97.97-99.77] | 98.91  [97.84-99.97] | 99.17  [97.52-100.0] | 97.24  [92.02-100.0] |
| GR | ACC (%)  [95% CI] | 88.47  [86.07-90.87] | 89.15  [86.41-91.89] | 86.67  [81.18-92.17] | 87.24  [78.05-96.43] |
|  | Sensitivity (%)  [95% CI] | 82.55  [78.45-86.65] | 81.12  [76.13-86.11] | 88.23  [80.59-95.88] | 81.74  [68.66-94.82] |
|  | Specificity (%)  [95% CI] | 94.36  [91.94-96.79] | 97.1  [94.98-99.22] | 85.45  [77.71-93.18] | 100.0  [100.0-100.0] |
|  | PPV (%)  [95% CI] | 93.59  [90.9-96.27] | 96.5  [93.95-99.06] | 83.0  [74.26-91.74] | 100.0  [100.0-100.0] |
|  | NPV (%)  [95% CI] | 84.46  [80.78-88.15] | 83.93  [79.62-88.24] | 90.03  [83.42-96.63] | 71.06  [51.47-90.66] |

Note: NPV, negative predictive value; PPV, positive predictive value; Total, collection of patients in all validation cohorts; VC1, validation cohort1; VC2, validation cohort2; VC3, validation cohort3; ACC, overall accuracy; pCR, pathological complete response; GR, good response; ACC, accuracy.

| **ID** | **Model tag** | **Feature names** | **Type** |
| --- | --- | --- | --- |
| R1 | Rm | original_firstorder_RobustMeanAbsoluteDeviation-T2 | firstorder |
| R2 | Rm | wavelet-HL_firstorder_RootMeanSquared-ADC | wavelet |
| R3 | Rm | wavelet-HH_glcm_DifferenceAverage-T2 | wavelet |
| R4 | Rm | wavelet-HH_glcm_Idm-T2 | wavelet |
| R5 | Rm | wavelet-LL_glszm_ZonePercentage-ADC | wavelet |
| R6 | Rm,RPm | wavelet-LL_glszm_SmallAreaEmphasis-ADC | wavelet |
| R7 | Rm,RPm | log-sigma-5-0-mm-3D_glcm_Correlation-ADC | wavelet |
| R8 | RPm | original_glcm_Imc2-T2 | texture |
| R9 | RPm | log-sigma-5-0-mm-3D_glcm_Idmn-T2 | texture |
| R10 | RPm | original_glrlm_LongRunEmphasis-ADC | texture |
| R11 | RPm | original_gldm_DependenceVariance-ADC | texture |
| R12 | RPm | wavelet-LL_glcm_Imc2-ADC | texture |
| P1 | Pm | AreaShape_Zernike_2_0-max | shape |
| P2 | Pm | Texture_AngularSecondMoment_Hematoxylin_4_45-25percent | texture |
| P3 | Pm,RPm | AreaShape_Zernike_8_4-max | shape |
| P4 | Pm | AreaShape_Zernike_8_6-min | shape |
| P5 | Pm | AreaShape_Solidity-25percent | shape |
| P6 | Pm | AreaShape_Zernike_5_1-std | shape |
| P7 | Pm,RPm | Texture_Entropy_Hematoxylin_4_0-75percent | texture |
| P8 | RPm | AreaShape_Zernike_2_0-mean | shape |
| P9 | RPm | AreaShape_Zernike_6_4-75percent | shape |
| P10 | RPm | AreaShape_Zernike_9_7-max | shape |
| P11 | RPm | AreaShape_MeanRadius-25percent | shape |

**Table S6.** Types of selected features

Note: -T2, extracted feature from T2 weighted sequence of MRI; -ADC, extracted feature from ADC sequence of MRI; -max, the maximum value of the features among all tiles from ROI of WSI; -min, the minimum value of the features among all tiles from ROI of WSI; -25percent, first quarter of feature value among all tiles from ROI of WSI; -75percent, last quarter of feature value among all tiles from ROI of WSI; R, radiomic features from MRI; P, pathomic features from biopsy WSI; Rm, radiomics model; Pm, pathomics model; RPm, radiopathomics model.
