# Supplementary material for: Multiparametric MRI and Whole Slide Image-Based Pretreatment Prediction of Pathological Response to Neoadjuvant Chemoradiotherapy in Rectal Cancer: A Multicenter Radiopathomic Study
Source: Ann Surg Oncol. 2020 Jul 29;27(11):4296–306. doi: 10.1245/s10434-020-08659-4 (PMC7497677; doi:10.1245/s10434-020-08659-4)
Supplement: Supplementary file 2 — Supplementary material 2 (DOCX 39 kb) [file 10434_2020_8659_MOESM2_ESM.docx]

**SUPPLEMENTARY INFORMATION**

**I. Neoadjuvant chemo-radiotherapy treatment**

**II. Pathological treatment response evaluation**

**III. Annotation of MRI**

**IV. Features extraction from mp-MRI**

**V. Features extraction from WSI**

**VI. Feature selection and training strategy of radiopathomics model**

**VII. Feature extraction, selection and predictive signature construction**

**I. Neoadjuvant chemo-radiotherapy treatment**

All LARC patients received neoadjuvant radiotherapy concurrently given 5-fluorouracil based chemotherapy orally or intravenously. In some instances, patients also received 5-fluorouracil based neoadjuvant chemotherapy combined with or without oxaliplatin before or/and after the nCRT, and the treatment cycles were at physician discretion. Intensity-Modulated Radiation Therapy or Volumetric Modulated Arc Therapy was administered using Elekta Synergy or Varian Rapidarc treatment system. The neoadjuvant radiotherapy was delivered at 25 fractions of 2 Gy (gross tumor volume, GTV) and 1.8 Gy (clinical target volume, CTV) radiation. A total dose of 50 Gy (GTV)/45 Gy (CTV) radiation was administered per day (weekday, day 1 to day 5) over a period of 5 weeks. The total mesorectal excision (TME) surgery was performed 4-8 weeks after the completion of nCRT, based on standard TME operation protocol.

**II. Pathological treatment response evaluation**

Surgical specimens with hematoxylin-eosin staining (H&E) staining was performed under a standard protocol in each participating hospital. A central pathological laboratory was set up to review and score the pathological treatment response at Guangdong Institute of Gastroenterology, the Sixth Affiliated Hospital of Sun Yat-sen University. The four-tier the American Joint Committee on Cancer/College of American Pathologists (AJCC/CAP) tumor regression grade (TRG) system was employed to evaluate the treatment response according to the volume of residual tumor cells: TRG 0 (complete response), no remaining viable cancer cells; TRG 1 (moderate response), only small cluster or single cancer cells remaining; TRG 2 (minimal response), residual cancer remaining, but with predominant fibrosis; TRG 3 (poor response), minimal or no tumor kill with extensive residual cancer [1]. AJCC/CAP TRG category was defined independently by two expert gastroenterology pathologists, who were blinded to clinicopathological and survival information. Disagreement in AJCC/CAP TRG classifications between the two pathologists would be judged by the third one.

**III. Annotation of MRI**

ROIs were outlined along the contour of the tumor mass on T2WI and DWI containing the surrounding chords and burrs. If a low, mixed-intensity or any other non-normal rectal wall signal was detected in the tumor zone on T2WI (abnormal signal), the ROIs would be drawn with contouring of the abnormal signal region. ROIs were placed on the high signal intensity region on DWI. Care was taken to avoid the magnetic susceptibility artifact on DWI. ADC map was generated by a single exponential model, and ROI on DWI was automatically copied to the ADC diagram. Four radiologists (1 from each participating hospital) with at least 10 years' experience in rectal MR imaging were responsible for drawing and evaluating tumor masking. Inter- and intra-observer reproducibility of tumor segmentation and radiomic features extraction were initially analyzed with the T2WI data of 20 randomly selected patients for ROI-based radiomic features generation in a blinded fashion by these 4 radiologists.

**IV. Features extraction from mp-MRI**

Since the intensity values of MR images distribute widely, we used z-score normalization to make the image intensities have the properties of a standard normal distribution with $\mu=1$ and $\sigma=0$, where $\mu$ is the mean value of the images, and $\sigma$ is the standard deviation. The normalized values (also called z scores) of the image intensities (*x*) were calculated as follows:

$$z= \frac{x-\mu}{\sigma}$$

After z-score normalization of image pixel intensities, a total of 702 quantitative imaging features including (i) 18 first-order features, (ii) 14 morphology features, (iii) 68 textural features, (iv) 344 wavelet features, and (v) 258 log feature based on 1,3,5 filter size, were extracted respectively for T2 images, ADC maps using corresponding ROIs. Group1 features were the calculated quantified tumor intensity characteristics of first-order from the histogram of all tumor intensities. Group2 features were based on tumor morphological characteristics (i.e., diameter, axis length, elongation, etc.). Group3 comprised textual features based on the quantification of intratumoral heterogeneity (i.e., differences in texture observed within the tumor volume); Group4 expanded the image to the frequency domain to describe the texture features of tumors by changing the original image via wavelet transform, focusing on the various frequency scales and different feature orientations within the tumor volume. Group5 was calculated from origin images with log filters. In the experiments, all the features were all calculated using two-dimensional analysis and averaged for all slices within the three-dimensional tumor volume.

In the experiments, all MRI sequences radiomic features were generated by Pyradiomics (Version 2.11, https://github.com/Radiomics/pyradiomics) [2] using Python (Version 3.6.5). Since there is a detailed mathematical definition of the adopted iconographic features on the Pyradiomics website, we have not repeated the total description in the supporting materials. The whole mathematical definitions of imaging features can be found on PyRadiomics web pages of radiomic features definition (https://pyradiomics.readthedocs.io/en/latest/radiomics.html). Specific feature names were provided to help readers locate specific features, the specific features’ names in our study were listed in the following two tables, which were consistent with the feature name defined in pyradiomics **(Table A1)**.

**Table A1.** The names of all the categories of features extracted by pyradiomics in the study

| Feature Groups (*N*) | Names |
| --- | --- |
| First order statistics  (*N*=18) | 1. firstorder_10Percentile 2. firstorder_90Percentile 3. firstorder_Energy 4. firstorder_Entropy 5. firstorder_InterquartileRange 6. firstorder_Kurtosis 7. firstorder_Maximum 8. firstorder_MeanAbsoluteDeviation 9. firstorder_Mean 10. firstorder_Median 11. firstorder_Minimum 12. firstorder_Range 13. firstorder_RobustMeanAbsoluteDeviation 14. firstorder_RootMeanSquared 15. firstorder_Skewness 16. firstorder_TotalEnergy 17. firstorder_Uniformity 18. firstorder_Variance |
| Shape  (*N*=14) | 1. shape_Elongation 2. shape_Flatness 3. shape_LeastAxisLength 4. shape_MajorAxisLength 5. shape_Maximum2DDiameterColumn 6. shape_Maximum2DDiameterRow 7. shape_Maximum2DDiameterSlice 8. shape_Maximum3DDiameter 9. shape_MeshVolume 10. shape_MinorAxisLength 11. shape_Sphericity 12. shape_SurfaceArea 13. shape_SurfaceVolumeRatio 14. shape_VoxelVolume |
| GLCM  (*N*=22) | 1. glcm_Autocorrelation 2. glcm_JointAverage 3. glcm_ClusterProminence 4. glcm_ClusterShade 5. glcm_ClusterTendency 6. glcm_Contrast 7. glcm_Correlation 8. glcm_DifferenceAverage 9. glcm_DifferenceEntropy 10. glcm_DifferenceVariance 11. glcm_JointEnergy 12. glcm_JointEntropy 13. glcm_Imc1 14. glcm_Imc2 15. glcm_Idm 16. glcm_Idmn 17. glcm_Id 18. glcm_Idn 19. glcm_InverseVariance 20. glcm_MaximumProbability 21. glcm_SumEntropy 22. glcm_SumSquares |
| GLRLM  (*N*=16) | 1. glrlm_GrayLevelNonUniformity 2. glrlm_GrayLevelNonUniformityNormalized 3. glrlm_GrayLevelVariance 4. glrlm_HighGrayLevelRunEmphasis 5. glrlm_LongRunEmphasis 6. glrlm_LongRunHighGrayLevelEmphasis 7. glrlm_LongRunLowGrayLevelEmphasis 8. glrlm_LowGrayLevelRunEmphasis 9. glrlm_RunEntropy 10. glrlm_RunLengthNonUniformity 11. glrlm_RunLengthNonUniformityNormalized 12. glrlm_RunPercentage 13. glrlm_RunVariance 14. glrlm_ShortRunEmphasis 15. glrlm_ShortRunHighGrayLevelEmphasis 16. glrlm_ShortRunLowGrayLevelEmphasis |
| GLSZM  (*N*=16) | 1. glszm_GrayLevelNonUniformity 2. glszm_GrayLevelNonUniformityNormalized 3. glszm_GrayLevelVariance 4. glszm_HighGrayLevelZoneEmphasis 5. glszm_LargeAreaEmphasis 6. glszm_LargeAreaHighGrayLevelEmphasis 7. glszm_LargeAreaLowGrayLevelEmphasis 8. glszm_LowGrayLevelZoneEmphasis 9. glszm_SizeZoneNonUniformity 10. glszm_SizeZoneNonUniformityNormalized 11. glszm_SmallAreaEmphasis 12. glszm_SmallAreaHighGrayLevelEmphasis 13. glszm_SmallAreaLowGrayLevelEmphasis 14. glszm_ZoneEntropy 15. glszm_ZonePercentage 16. glszm_ZoneVariance |
| GLDM  (*N*=14) | 1. gldm_DependenceEntropy 2. gldm_DependenceNonUniformity 3. gldm_DependenceNonUniformityNormalized 4. gldm_DependenceVariance 5. gldm_GrayLevelNonUniformity 6. gldm_GrayLevelVariance 7. gldm_HighGrayLevelEmphasis 8. gldm_LargeDependenceEmphasis 9. gldm_LargeDependenceHighGrayLevelEmphasis 10. gldm_LargeDependenceLowGrayLevelEmphasis 11. gldm_LowGrayLevelEmphasis 12. gldm_SmallDependenceEmphasis 13. gldm_SmallDependenceHighGrayLevelEmphasis 14. gldm_SmallDependenceLowGrayLevelEmphasis |
| Wavelets  (*N*=344) | 1. wavelet-LH_* (86) 2. wavelet-HL_* (86) 3. wavelet-LL_* (86) 4. wavelet -HH_* (86) |
| Logs-  (*N=*258) | 1. Log-sigma-1.0_* (86) 2. Log-sigma-3.0_* (86) 3. Log-sigma-5.0_* (86) |

Note:

GLCM, Gray-level co-occurrence matrices; GLRLM, Gray-level run length matrix; GLSZM, Gray-level size zone matrix; GLDM, Gray level dependence matrix;

*: The abbreviated representation of feature types. Feature types contained First order statistics, GLCM, GLRLM, GLSZM and GLDM;

(n): Represents the total number of features represented by abbreviations.

**V. Features extraction from WSI**

ROIs on WSI were cut into 57$\pm$25 (width × height = 512×512) tiles for feature extraction by non-overlapping sampling at magnification levels 20X (0.5$\mu m/pixel$). A total of 154 features including pixel intensity, morphology, and nuclear texture for each ROI were extracted. Then, the features were aggregated across the case-level tiles by the mean, median, standard deviation, 25-quantiles of the values, and 75-quantiles of the values. In present study, the color channel separation of ROIs, nucleus segmentation, and feature calculation were implemented via modules named ‘UnmixColors’, ‘IdentifyPrimaryObjects’, ‘Measure ObjectIntensity’, ’MeasureObjectSizeShape’, and ’Measure Texture’ in the CellProfiler platform (Version 2.2.1, https://cellprofiler.org/).[3]

**VI. Feature selection and training strategy of radiopathomics model**

The model combined both radiomic features and pathomic features and collected radiopathomic features by feature selection to reveal the interaction between macro and micro information. To narrow the feature selection range and reduce feature redundancy, a coarse to shallow feature screening strategy was used.

In the initial screening stage, The eXtreme Gradient Boosting (XGBoost) [4] was utilized (https://github.com/dmlc/xgboost) as the framework for radiopathomics features. XGBoost's feature selection process was similar to a random forest. The entire model was composed of many decision trees. Each decision tree has multiple tree nodes. These nodes corresponded to the judgment conditions and weights of the feature input. The XGBoost tries different feature combinations through the traversal strategy in the training set. Each feature selected as a tree node will calculate the contribution to the final prediction result (Gain), which was the importance of features. The average gain of the feature was recorded, as the basis for feature selection. After the model training for feature selection in the training set was completed, a list of Gain corresponding to all selected features was obtained. Then, the list was sorted, and the top n % of features will be selected as the final selected modeling feature (in this article we chose the top 10%).

Next, the Spearman test was used to refine the feature screening results. The higher the correlation, the more similar the distribution of features on the sample combination was to the actual label distribution. Subsequently, the results were ranked in descending order of correlation values, and the characteristic variables of *P*<0.05 were excluded, which lacked significance. All feature screening operations were performed on the primary cohort.

Finally, the XGBoost framework combined with the results of feature selection to build models for training and prediction again. In order to limit the negative impact of hyper-parameter in comparative experiments, and examine the improvement of prediction results from the strategy of fusing mp-MRI and biopsy WSI under the same modeling conditions, we used the same model structure and hyper-parameters both in feature selection stage and modeling stage through all models. Gradient boosting tree (gbtree) was the basis of the predictive model, which was nonlinear, and the max depth of each gbtree was set to 12. The gama was 0.05, which was the minimum loss reduction required to make a further partition on a leaf node of the tree. Softmax was chosen as the objective function of the model. During the training phase, the rounds of boosts was set to 500. In order to get a better fit state during the training process, the learning rate was set to 0.01 and the model will terminate the training and save the weight of the model after 50 iterations in which the accuracy of the validation set stops increasing.

*Formula definitions:*

*F-1: Function of model prediction*

$Outputs=argmax(\frac{e^{\hat{y}_{i}}}{\sum_{j} e^{\hat{y}_{i}}})$, $Outputs$ was the final results of the model, which was categories. $\hat{y}_{i}$ was the prediction score of i-th category from the prediction model.

$\hat{y}_{i}=\sum_{k=1}^{K} f_{k}\left( x_{i} \right), f_{k}\mathcal{\in F}$, $\hat{y}_{i}$ is the prediction score of i-th category from the prediction model. $f_{k}$ was the function of the k-th tree, $i$ was the category order.

*F-2: Objective function of model training*

$Obj=\sum_{i=1}^{n} l\left( y_{i},\hat{y}_{i} \right)+\sum_{k=1}^{K} \Omega(f_{k})$;

$l\left( y_{i},\hat{y}_{i} \right)$ was the loss of model in training. $\Omega(f_{k})$ was the complexity of XGBoost.

*F-3: Importance function of features in the model:*

$Gain=\frac{1}{2}\left[ \frac{G_{L}^{2}}{H_{L}+\lambda}+\frac{G_{R}^{2}}{H_{R}+\lambda}-\frac{{(G_{L}+G_{R})}^{2}}{H_{L}+H_{R}+\lambda} \right]-\gamma$,

$G_{*}$: The sum of the first partial derivatives of the samples contained in the leaf node, which was a constant;

$H_{*}$: The sum of the second-order partial derivatives of the samples contained in leaf node, which was a constant.

**VII. Feature extraction, selection and predictive signature construction**

For radiomic features from mp-MRI, intra-class correlation coefficients (ICCs) were utilized for evaluating the intra- and inter-observer agreement in terms of feature extraction and avoiding the negative impact of manual segmentation on the extracted features. In the study, we firstly invited different doctors to sketch the tumor area in the MRI as ROI multiple times at different time points, and extracted the same category of image features from ROI. We interpreted an ICC of 0.81-1.00 as almost perfect agreement, 0.61-0.80 as substantial agreement, 0.41-0.60 as moderate agreement, 0.21-0.40 as fair agreement, and 0-0.20 as poor or no agreement.[5] Then, satisfactory inter- and intraobserver reproducibility of tumor masking and radiomic feature extraction was achieved with ICC > 0.6 both among the masks. For pathomic features from WSI, all of our pathomic features were extracted from the ROI of the nucleus area, which was automatically segmented by imaging processing method using CellProfile. [3]

For features pool combining radiomic features with pathomic features, 13 features were selected. For radiomic features alone, we selected 7 features according to our feature screening criteria. For pathomic features alone, 7 features were selected. (**Table A2**). In the Table A2, R (7 features) represented selected features from all radiomics features, which inputs of XGBoost only contained all radiomic features during feature selection. P (7 features) represented selected features from all pathomic features, which inputs of XGBoost only contained all pathomic features during feature selection. RP (14 features) represented selected features from the integration of all radiomic features and pathomic features. The radiomic features set only contained features from mp-MRI and the pathomic features set only contained features from WSI. The integration of all radiomic features and pathomic features included both radiomic features and pathomic features. There was a statistically significant correlation between selected features and 4-category AJCC/CAP TRG (*P*<0.05) **(Figure S5)**.

**Table A2.** Modeling features

| ID | Features | Type |
| --- | --- | --- |
| R1 | original_firstorder_RobustMeanAbsoluteDeviation-T2 | firstorder |
| R2 | wavelet-HL_firstorder_RootMeanSquared-ADC | wavelet |
| R3 | wavelet-HH_glcm_DifferenceAverage-T2 | wavelet |
| R4 | wavelet-HH_glcm_Idm-T2 | wavelet |
| R5 | wavelet-LL_glszm_ZonePercentage-ADC | wavelet |
| R6, RP1 | wavelet-LL_glszm_SmallAreaEmphasis-ADC | wavelet |
| R7, RP2 | log-sigma-5-0-mm-3D_glcm_Correlation-ADC | wavelet |
| P1 | AreaShape_Zernike_2_0-max | shape |
| P2 | Texture_AngularSecondMoment_Hematoxylin_4_45-25percent | texture |
| P3, RP3 | AreaShape_Zernike_8_4-max | shape |
| P4 | AreaShape_Zernike_8_6-min | shape |
| P5 | AreaShape_Solidity-25percent | shape |
| P6 | AreaShape_Zernike_5_1-std | shape |
| P7, RP4 | Texture_Entropy_Hematoxylin_4_0-75percent | texture |
| RP5 | original_glcm_Imc2-T2 | texture |
| RP6 | log-sigma-5-0-mm-3D_glcm_Idmn-T2 | texture |
| RP7 | original_glrlm_LongRunEmphasis-ADC | texture |
| RP8 | original_gldm_DependenceVariance-ADC | texture |
| RP9 | wavelet-LL_glcm_Imc2-ADC | texture |
| RP10 | AreaShape_Zernike_2_0-mean | shape |
| RP11 | AreaShape_Zernike_6_4-75percent | shape |
| RP12 | AreaShape_Zernike_9_7-max | shape |
| RP13 | AreaShape_MeanRadius-25percent | shape |
| Note:  -T2, feature extraction from T2 weighted sequence of MRI; -ADC, feature extraction from ADC sequence of MRI; -max, the maximum value of the features among all tiles from ROI of WSI; -min, the minimum value of the features among all tiles from ROI of WSI; -25percent, first quarter value of features among all tiles from ROI of WSI; -75percent, last quarter value of features among all tiles from ROI of WSI; R, features for building radiomcis model; P, features for building pathomics model; RP, features for building radiopathomics model. | | |

**References**

1. Edge SB, Compton CC. The American Joint Committee On Cancer: The 7Th Edition of the Ajcc Cancer Staging Manual and the Future of Tnm. *Ann Surg Oncol*. 2010; 6: 1471-4.

2. Jjm VG, Fedorov A, Parmar C et al. Computational Radiomics System to Decode the Radiographic Phenotype. *Cancer Res*. 2017; 21: e104.

3. Carpenter AE, Jones TR, Lamprecht MR et al. Cellprofiler: Image Analysis Software for Identifying and Quantifying Cell Phenotypes. *Genome Biol*. 2006; 10: R100.

4. Chen T, Guestrin C. Xgboost: A Scalable Tree Boosting System. *Proceedings of the 22nd ACM SIGKDD International Conference on Knowledge Discovery and Data Mining*. San Francisco, California, USA: ACM, 2016: 785-94.

5. Vecchio FM, Valentini V, Minsky BD et al. The Relationship of Pathologic Tumor Regression Grade (Trg) and Outcomes After Preoperative Therapy in Rectal Cancer. *International Journal of Radiation Oncology*Biology*Physics*. 2005; 3: 752-60.
